# Fibroblasts’ secretome from calcified and non-calcified dermis in *Pseudoxanthoma elasticum* differently contributes to elastin calcification

**DOI:** 10.1038/s42003-024-06283-6

**Published:** 2024-05-16

**Authors:** Francesco Demetrio Lofaro, Sonia Costa, Maria Luisa Simone, Daniela Quaglino, Federica Boraldi

**Affiliations:** Department of Life Sciences, University of Modena and Reggio Emilia, Modena, Italy

**Keywords:** Skin diseases, Pathogenesis

## Abstract

*Pseudoxanthoma elasticum* (PXE) is a rare disease characterized by ectopic calcification, however, despite the widely spread effect of pro/anti-calcifying systemic factors associated with this genetic metabolic condition, it is not known why elastic fibers in the same patient are mainly fragmented or highly mineralized in clinically unaffected (CUS) and affected (CAS) skin, respectively. Cellular morphology and secretome are investigated in vitro in CUS and CAS fibroblasts. Here we show that, compared to CUS, CAS fibroblasts exhibit: a) differently distributed and organized focal adhesions and stress fibers; b) modified cell-matrix interactions (i.e., collagen gel retraction); c) imbalance between matrix metalloproteinases and tissue inhibitor of metalloproteinases; d) differentially expressed pro- and anti-calcifying proteoglycans and elastic-fibers associated glycoproteins. These data emphasize that in the development of pathologic mineral deposition fibroblasts play an active role altering the stability of elastic fibers and of the extracellular matrix milieu creating a local microenvironment guiding the level of matrix remodeling at an extent that may lead to degradation (in CUS) or to degradation and calcification (in CAS) of the elastic component. In conclusion, this study contributes to a better understanding of the mechanisms of the mineral deposition that can be also associated with several inherited or age-related diseases (e.g., diabetes, atherosclerosis, chronic kidney diseases).

## Abstract (web summary)

An in vitro study on fibroblasts from clinically affected or unaffected skin of patients with Pseudoxanthoma elasticum indicates that local microenvironment guides the level of matrix remodeling and the extent of elastic fiber calcification.

## Introduction

Ectopic calcification (EC), i.e., deposition of calcium salts within soft connective tissues, occurs in both acquired and genetic diseases, causing significant morbidity and mortality, and can be classified in metastatic or dystrophic if associated with elevated serum levels of calcium/phosphate or with damaged/diseased tissues, respectively^1^.

Pathological calcification is an active process in which different cell types (e.g., vascular smooth muscle cells, endothelial cells, fibroblasts) regulate mineral nucleation/growth through the production and secretion of an altered ratio of pro- and anti-calcifying molecules^2–4^ which have been already extensively investigated, nevertheless, pathways and pathogenic mechanisms of EC are still partially known.

Studies performed on *Pseudoxanthoma elasticum* (PXE), a rare genetic disorder characterized by EC^5^, demonstrated that calcification specifically affects elastic fibers in certain areas of organs/systems (e.g., skin, cardiovascular system, eyes)^6,7^. Investigations on blood samples and/or on fibroblasts from PXE patients, compared to healthy subjects, revealed that mineralization is associated with: mild chronic oxidative stress^8–12^, altered expression levels, and/or activity of pro- and anti-calcifying molecules (e.g., tissue-non-specific alkaline phosphatase, matrix Gla protein, inorganic pyrophosphate)^13–15^ and of matrix metalloproteinases (MMP) 2 and 9^16^, activation of transforming growth factor-beta (TGF-β)/bone morphogenetic protein (BMP) pathway^17–19^, and mitochondrial bioenergetic abnormalities^20,21^. However, these findings, obtained by comparing samples from PXE and from healthy subjects, are not sufficient to clarify how, in PXE patients, only some elastic fibers become calcified even within the same tissue and why their amount is highly variable depending on the organ/system, despite the fact that the disease is considered an inherited metabolic disorder releasing soluble factors that should reach and affect whole soft connective tissues^22^.

Interestingly, it was recently demonstrated in PXE patients that in clinically unaffected skin (CUS; absence of papules and/or skin laxity) elastic fibers were altered, but calcification was a rare finding, in contrast abundant mineralization was typically observed in clinically affected skin (CAS; presence of papules and/or skin laxity)^18^.

Composition and organization of the extracellular matrix (ECM) determine how mesenchymal cells interact with and respond to their microenvironment^23^, therefore, changes in ECM modify tissue mechanical properties (i.e., matrix stiffness)^24^, and modulate cellular phenotype and function^25^. Several years ago it was demonstrated that PXE fibroblasts cultured from CAS had a different growth capacity compared to cells from CUS, suggesting that phenotypic differences may occur between CUS and CAS cells even in the same patient^26^.

Therefore, to better understand the phenotype of CUS and CAS fibroblasts and to get new light on their pathogenic role in the calcification process, we investigated: (i) the cellular morphology by light microscopy; (ii) the capacity to interact with the collagen substrate by confocal microscopy and by gel retraction assay; (iii) the protein profile of the secretome (i.e., proteins released by living cells in the culture medium) by liquid chromatography with tandem mass spectrometry (LC-MS/MS); (iv) the degradative potential and in vitro calcification by zymography and Alizarin red assay, respectively; (v) the expression of decorin and of high-mobility group box1 (HMGB1) by Western blot (WB) and of perlecan by dotblot; (vi) the effect of heparan sulfate and chondroitin sulfate on the in vitro mineralization of elastin fibrils; (vii) the expression of *BMP-2* by quantitative real‐time‐polymerase chain reaction (qRT-PCR).

## Results and discussion

### CUS and CAS fibroblasts show changes in cell shape and ECM-cell interactions

**Fig. 1:**
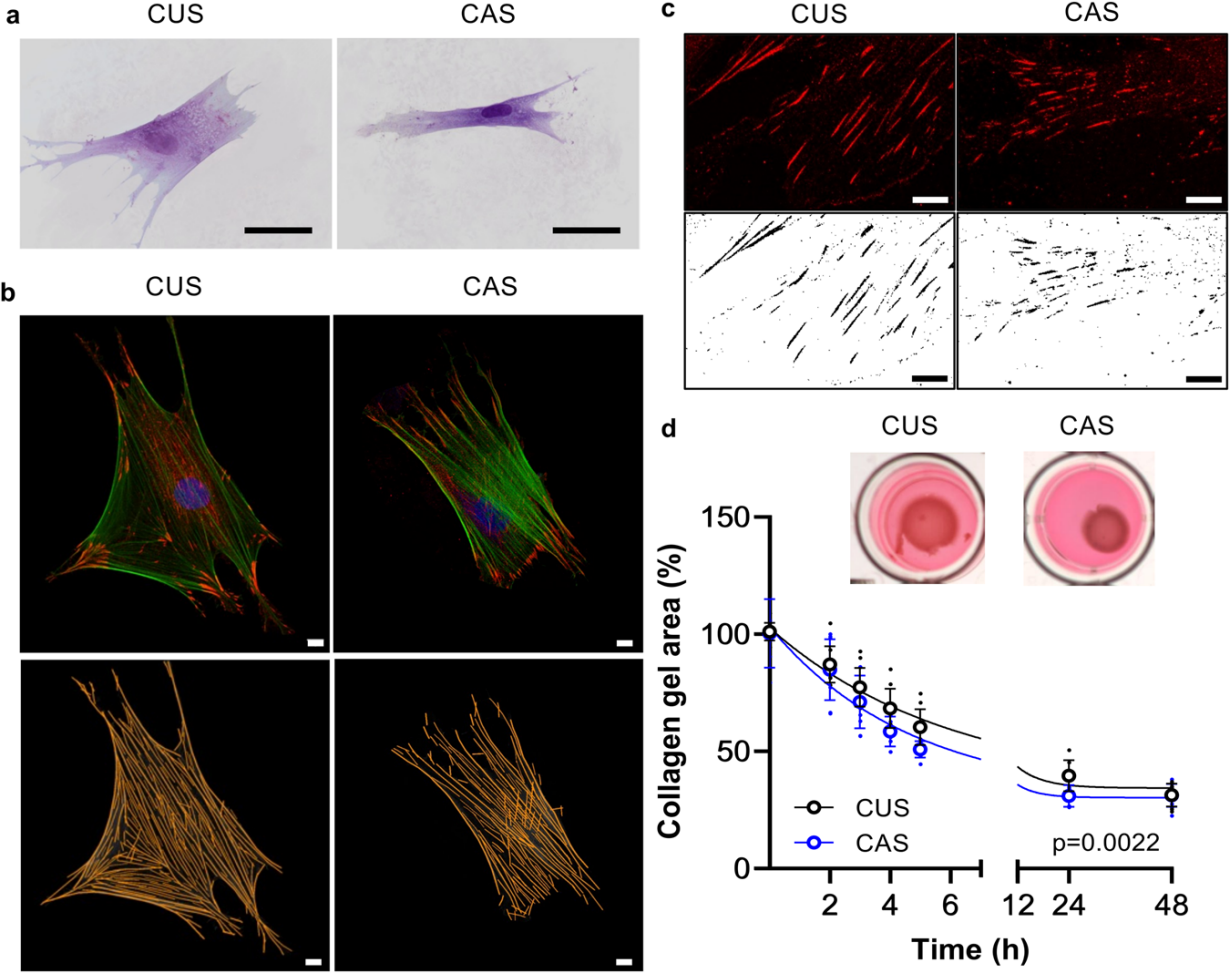
Cell shape and ECM-cell interactions. **a** Representative image of CUS and CAS fibroblasts seeded on a collagen coating. Scale bar: 50 µm. **b** Representative images of focal adhesions and of F-actin stress fibers in CUS and CAS fibroblasts. To reveal FAs and SFs, cells were labeled with anti-paxillin antibody (red) and with phalloidin (green), respectively. Scale bar: 10 µm. **c** Representative images of FAs in CUS and CAS fibroblasts. Scale bar: 10 µm. **d** Representative images of collagen gels retracted by fibroblasts isolated from CUS and CAS. Gel retraction is expressed as percentage of the initial collagen gel area. Data represent means ± SD of measures from three independent experiments performed in triplicate on cell lines kept separate (*n* = 3 biological replicates for CUS and *n* = 3 biological replicates for CAS for each time point).

It is known that different chemical/physical characteristics of the ECM and the interactions between cells and ECM influence cellular morphology^27,28^. Figure 1a shows CUS and CAS fibroblasts cultured on a collagen substrate to better highlight cell-matrix interactions. CUS cells were larger and flattened, whereas CAS fibroblasts showed a spindle-shaped and elongated phenotype. Area, perimeter, aspect ratio, and circularity were quantified by ImageJ software. Consistent with the observed differences in cell morphology, most shape descriptors were significantly different between cultured CUS and CAS fibroblasts (Table 1).

**Table 1:** Morphometric analysis performed on cells, stress fibers, and focal adhesions

Parameters	CUS	CAS	*p* value
**Morphometric descriptor of cell shape**			
Area (μm^2^)	2168 ± 1000	1821 ± 721	*p* < 0.001
Perimeter (μm)	494 ± 301	461 ± 195	n.s.
Aspect ratio (a.u.)	3.02 ± 1.52	4.16 ± 2.35	*p* < 0.0001
Circularity (0–1) (a.u.)	0.17 ± 0.15	0.13 ± 0.10	*p* < 0.0001
**Morphological characterization of stress fibers**			
Lenght (μm)	106.1 ± 35.29	98.76 ± 32.13	*p* < 0.0001
Width (μm)	2.67 ± 0.66	2.76 ± 0.69	*p* < 0.0001
**Morphological characterization of focal adhesions**			
Area (μm^2^)	1.43 ± 0.28	1.33 ± 0.37	*p* < 0.0001
Perimeter (μm)	7.28 ± 1.30	7.42 ± 1.73	n.s.
Aspect ratio (a.u.)	3.58 ± 0.74	3.13 ± 0.68	*p* < 0.0001

Values represent means ± SD. *n.s.* no significant.

It is known that cell shape is related to changes in the cytoskeleton, a cytoplasmic interconnected network of protein filaments, and in the physical interactions between cells and ECM^29^. Major components of the cytoskeleton are stress fibers (SFs) composed of contractile actin bundles, which are usually connected at both ends to focal adhesions (FAs) represented by large macromolecular multiprotein assemblies.

By confocal microscopy, both SFs (Fig. 1b) and FAs were analyzed in all cell lines (Fig. 1b, c). Morphological parameters indicated that SFs were significantly shorter and thicker in CAS than in CUS fibroblasts (Table 1). The production of thick bundles of actin filaments is driven by ATP hydrolysis, possibly indicating that altered energy metabolism in PXE^21,30^ can impact on the cytoskeletal organization and on mechano-transduction signaling^31,32^.

Labeling of cells for paxillin, a typical component of FAs essential to their formation^33^, revealed, by confocal microscopy, that paxillin was mostly concentrated at the end of the long axis in CAS cells, whereas it was homogeneously distributed over the cell body in CUS fibroblasts (Fig. 1c). Furthermore, size and shape descriptors indicated that the area of FAs and the aspect ratio (length/width) of adhesions were significantly smaller in CAS than in CUS fibroblasts (Table 1). To be noted that these changes were not related to altered paxillin gene expression since CAS/CUS fold change was 1.00 ± 0.07, as evaluated by qRT-PCR.

However, the ratio between the area of FAs and the cell area in two cellular types (ratio_CAS_ = 0.074 ± 0.002 *vs*. ratio_CUS_ = 0.063 ± 0.002, *p* < 0.0001) indicates that the contact surface area in CAS fibroblasts is greater than that of CUS cells (i.e., an increase of 17%), suggesting a higher cellular tension associated with thicker SFs in CAS compared to CUS cells.

SFs, through FAs, connect the cytoskeleton to the ECM and their modification can trigger a different ECM remodeling^34^. An in vitro collagen gel contraction assay was used as a simple 3D model suitable for studying cell-ECM interactions and cellular contractility ^35^. Since contractile forces depend on both the number of cells embedded in the collagen gel and the collagen concentration^36^, these two parameters were kept constant in these experiments. Cell-populated collagen gels were physically detached from the dish soon after polymerization, leaving the collagen gel floating in the culture medium. Figure 1d shows the reduction of the gel area over time. The contraction of the free-floating gel occurred rapidly (i.e., few hours) for all cell lines, however, CAS fibroblasts generated greater contractile forces than CUS fibroblasts, as shown by the greater reduction of the collagen gel size (Fig. 1d). After 24 hours of culture, the gel size did not exhibit further changes (Fig. 1d). Indeed, the stress fibers disassemble over time since the isometric tension, typical of collagen gels attached to the dish, is lost in the free-floating gels^37,38^. Although previous studies reported a low ability of PXE fibroblasts to retract the collagen gel^26,39^, it is important to underline that, in the past, the comparison was made between fibroblasts from PXE patients and from healthy individuals, whereas in the present study fibroblasts were analyzed from CUS and CAS areas within the same PXE patients.

In summary, results indicate that CUS and CAS fibroblasts (i) exhibit a different morphology in standard culture conditions; (ii) are characterized by a different cell-tension property and cell-matrix interactions^40^; (iii) show changes in the distribution and organization of FAs and SFs. Indeed, the generation of SFs-mediated tension in fibroblasts can activate latent-transforming-growth factor β1, a multifunctional cytokine acting on ECM-homeostasis, on BMP-mediated osteogenic signaling as well as on cellular redox metabolism^41^. It could be suggested that, also in vivo, modifications in cell-matrix interactions and in the cytoskeletal organization can influence cell behavior and that changes in ECM may alter the stiffness of the tissues and the response of cells to mechanical stimuli (e.g., stretching, tensile strength), consistent, for instance, with the prevalence of skin alterations in flexural areas.

### ECM molecules are differentially expressed by fibroblasts in CUS and CAS secretomes

Proteins released by living cells in the surrounding extracellular space (i.e., secretome) play a key role in regulating several physio-pathological processes, including EC^4,42^. Therefore, to the best of our knowledge, for the first time, the secretome of fibroblasts from three PXE patients was investigated by comparing CUS and CAS data within each patient to avoid inter-individual variability.

**Fig. 2:**
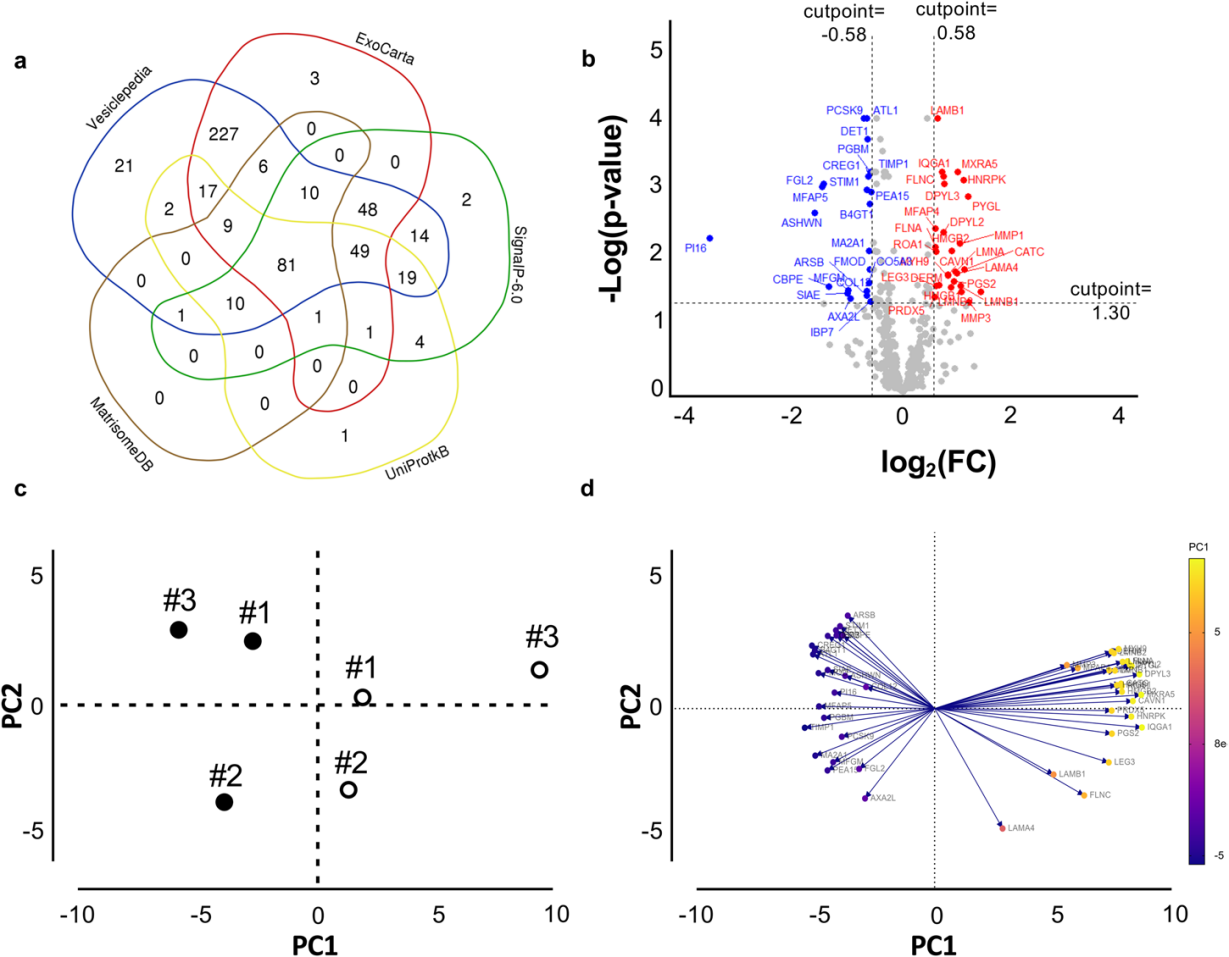
Fibroblasts’ secretome. **a** A Venn diagram shows the distribution of proteins identified by LC-MS/MS in different databases. **b** Up- (red) and down- (blue) regulated, or unchanged (gray) proteins are shown in the Volcano plot. The X-axis represents the log_2_ values of the fold change (FC) observed for each protein, and the *Y* axis depicts the log_10_ values of the *p* value of the significance tests between replicates for each protein. Proteins with log_2_ FC (CAS/CUS) > 0.58 (red) or <−0.58 (blue) and with a *p* value < 0.05 are displayed. **c** Principal component analysis (PCA) shows the spatial distribution of secretomes comparing CUS (filled circles) and CAS (empty circles) data. Each dot represents the cell lines from three PXE patients. **d** Loading plot of data shows the variables that contribute the most to spatial distribution observed in **c**. The length and the direction of vectors indicate how each ratio readout contributes to the two principal components in the plot.

By mass spectrometry, 536 proteins were identified with at least one unique peptide (Supplementary Data 1). To provide a spatial context (i.e., polypeptide localization), proteins identified by LC-MS/MS were searched against the extracellular protein genes annotated in five public databases. 98% of proteins (i.e., 526/536) were found in at least one database: 514 in Vesiclepedia, 452 in ExoCarta, 240 in SignalP 6.0, 194 in UniprotkB and 118 in MatrisomeDB (Fig. 2a and Supplementary Data 1). The proteins found in this last database have been indicated as collagens, proteoglycans, glycoproteins, secreted factors, ECM-affiliated proteins, and ECM regulators.

To compare the relative abundance of secreted proteins from CUS and CAS fibroblasts, we used a label-free quantification based on the measure of precursor ion intensities of proteins that, for this purpose, were identified with at least two peptides (i.e., 486/536) to increase the accuracy of measurements^43,44^.

Most proteins did not change between CUS and CAS, but 50/486 proteins were differentially expressed proteins (DEPs) between the two conditions (Fig. 2b and Supplementary Data 2), in particular 23 and 27 were significantly down- and upregulated in CAS compared to CUS, respectively.

The score plot highlights differences between secretomes of CUS and CAS fibroblasts (Fig. 2c). The variables contributing the most to this separation are shown in Fig. 2d.

### Potential lytic activity is higher in CAS than in CUS secretome

By LC-MS/MS analysis 58/486 proteases (n. 37) and protease inhibitors (n. 21) were quantified, of which 6/58 were DEPs (Supplementary Data 3).

Among differentially expressed proteases, an interesting role can be exerted by the proprotein convertase subtilisin kexin type 9 (PCSK9). Previous studies demonstrated that PCSK9 in cell lysates of PXE fibroblasts was significantly increased compared to healthy cells grown in serum-free condition^30^. In our experimental model a decrease of PCSK9 in CAS compared with CUS secretome was found. This molecule plays a key role in the regulation of LDL-cholesterol and of genes related to oxidative stress, inflammation, apoptosis^45,46^. Moreover, it is also involved in arterial medial and aortic valve mineralization^47,48^ and is overexpressed in smooth muscle cells (SMCs) cultured in a calcific environment^48^. Interestingly, the addition of recombinant human PCSK9 was shown ineffective on SMCs calcification, suggesting that is the intracellular PCSK9 able to promote a switch towards a pro-calcific phenotype^48^. Therefore, additional studies are foreseen to better investigate the possible role of the intracellular and secreted forms of PCSK9 in PXE and in the process of ectopic calcification.

Another interesting finding revealed the significant increase of MMP-1 and MMP-3 in CAS compared to CUS secretome. It is already known that PXE patients are characterized by higher circulating levels of MMP-2 and MMP-9 compared to healthy controls^16^, and that PXE fibroblasts, in comparison with healthy cells, show increased levels of MMP-1, MMP-2, and MMP-3^49,50^, which are known to be active also on elastic fibers^51^. Although elastic fiber degradation has already been suggested in the pathogenesis of ectopic calcification in PXE^52–54^, most importantly, this study shows that the degradation potential is different between CAS and CUS, thus creating microenvironments locally altering matrix homeostasis.

Within this context, we have also observed a reduced expression of the ADAMTS-like protein 1 (ATL-1) in the CAS secretome. This protein is a secreted molecule structurally resembling the ADAMTS family of proteases, although it does not seem to be involved in proteolytic activities but in regulating ECM assembly and/or cell-matrix interactions^55,56^. These data may support the effect of altered matrix composition and/or organization on different cell-matrix interactions observed in CAS and CUS fibroblasts.

Regarding protease inhibitors detected in the secretome (21/58), several exhibited a downward trend in CAS (Supplementary Data 3), of these, the metalloproteinase inhibitor 1 was significantly reduced in CAS compared to CUS.

Taken all together, these results highlight that the ratio between proteases and protease inhibitors, important to determine the extension of ECM degradation, is different between CUS and CAS secretomes. It is conceivable that the increase of proteases and the decrease of proteinase inhibitors in CAS may induce greater degradation/fragmentation of ECM molecules, which in turn promote/influence the mineralization process.

**Fig. 3:**
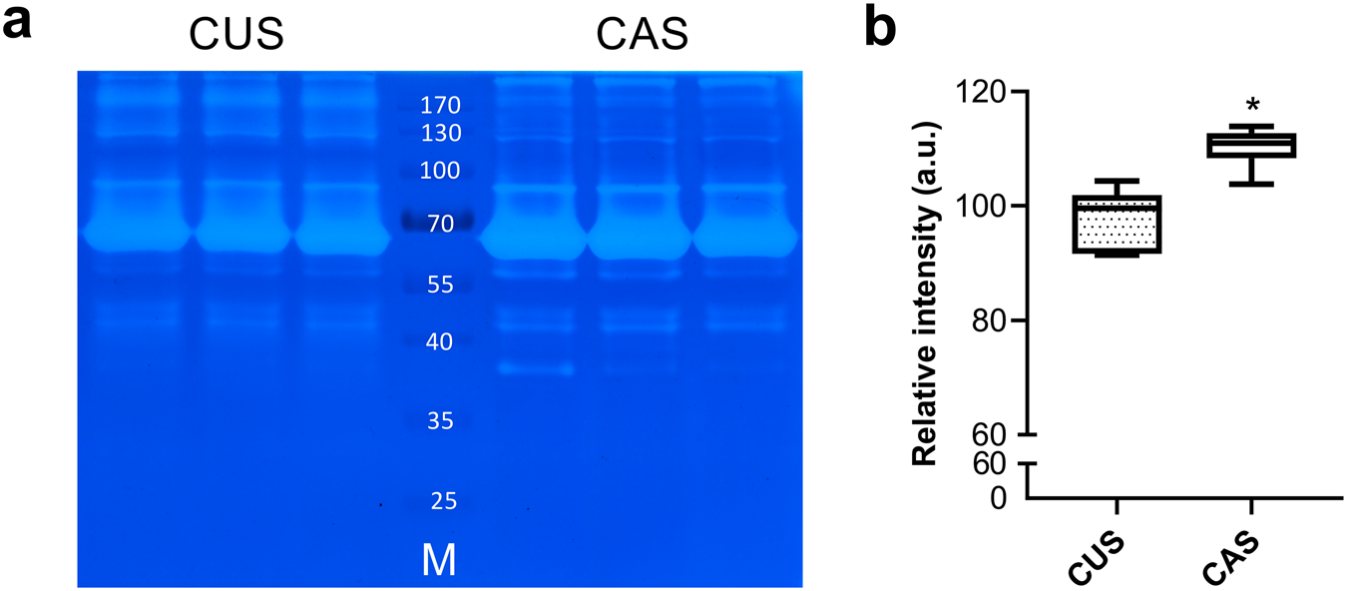
Potential lytic activity. **a** Representative collagen zymography gel loaded with the secretomes of CUS and CAS fibroblasts. M molecular weight (kDa) of standard proteins. **b** Densitometric analysis of all bands from CUS and CAS fibroblasts. The boxplots show the median (center line), interquartile range (bounds of box), the range of typical data values (whiskers). Data represent means ± SD of measures from three independent experiments performed in triplicate on cell lines kept separate (*n* = 3 biological replicates for CUS and *n* = 3 biological replicates for CAS). *p < 0.05.

Because the levels of enzyme expression do not always directly correspond to protease activity, the collagen zymography technique was used to assess the potential enzymatic activity^57^ of CUS and CAS secretomes. Figure 3a shows a representative gel from triplicate experiments in which several bands at different molecular weights were detectable in both CUS and CAS conditions. Densitometric analyses revealed that the potential lytic activity was significantly higher in CAS than in CUS (Fig. 3b).

**Fig. 4:**
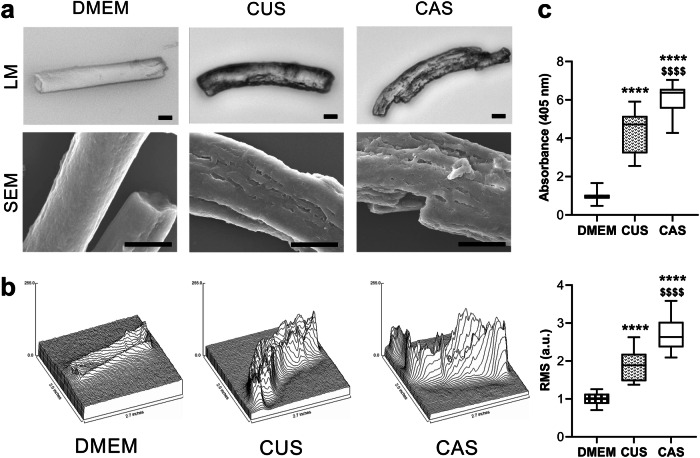
In vitro mineralization of elastin fibrils. **a** Representative images of elastin fibrils observed by light microscopy (LM) and by scanning electron microscopy (SEM). Elastin fibrils were incubated with standard culture medium (DMEM) or with medium conditioned by CUS or CAS fibroblasts. The experiment was performed with conditioned media obtained from each cell line. Scale bar: 10 μm. **b** Surface plot and root mean square (RMS) roughness of peaks and valleys measured on elastin fibrils incubated with DMEM or with medium conditioned by CUS or CAS fibroblasts. The boxplots show the median (center line), interquartile range (bounds of box), the range of typical data values (whiskers). For each experimental condition, 30 elastin fibrils were analyzed. **c** Insoluble elastin fibrils treated with standard culture medium (DMEM) or treated with medium conditioned by CUS or CAS fibroblasts were incubated with a calcifying medium for 72 hours and stained with Alizarin red to reveal calcium deposits. The boxplots show the median (center line), interquartile range (bounds of box), and the range of typical data values (whiskers). Data are from three independent experiments (*n* = 3 for DMEM, *n* = 9 for CUS, and *n* = 9 for CAS). ****p < 0.001 CUS or CAS *vs* DMEM; $$$$*p* < 0.0001 CAS vs. CUS.

To ascertain that the proteolytic potential in the CUS and CAS secretomes was able to degrade elastin, we used an in vitro model^58^ where fibrils made of insoluble elastin (ELN) were incubated with either standard culture medium (DMEM; absence of proteolytic enzymes) or with media conditioned by CUS or CAS fibroblasts. Morphological analyses, performed by light and scanning electron microscopy, showed that ELN fibrils incubated with DMEM exhibited a smooth surface, whereas ELN incubated with CUS and CAS conditioned media showed a damaged surface characterized by irregular cavities of different sizes (Fig. 4a). For a better evaluation of the surface of undigested and digested ELN fibrils, root mean square (RMS) roughness of peaks and valleys were measured. The mean value of RMS roughness shows a statistical difference among the three experimental conditions. In particular, ELN fibrillar surface changed from an overall smooth surface (i.e., DMEM condition) to a moderate (i.e., CUS condition) or to a marked roughness (i.e., CAS condition) (Fig. 4b). These results indicate that the proteolytic activity of the enzymes secreted in the culture medium by CUS and CAS fibroblasts is able to degrade at a different extent ELN, the major component of elastic fibers^59^.

It is well known that ELN has high affinity for calcium ions^60–62^ and from in vitro studies it was demonstrated that fragmentation/degradation of elastin fibrils is able to enhance the mineralization process^58^.

Therefore, ELN fibrils, undigested or digested with CUS or with CAS-conditioned media were then incubated with a calcifying medium for 72 hours and calcium concentration was quantified by Alizarin red assay. The degradation of ELN fibrils with CUS and CAS-conditioned media significantly increased the amount of calcium deposits by a factor of 2.3 and 4.0, respectively, when compared to undigested ELN. Interestingly, calcium levels were significantly higher after degradation with CAS- compared to CUS secretome (Fig. 4c).

It has been previously demonstrated that CAS fibroblasts have an increased proteolytic potential compared to healthy cells^16,49^ and that plasma and urine of PXE patients contain higher concentrations of desmosine and isodesmosine (biomarkers of elastin degradation) compared to healthy subjects^52,63,64^. Present data demonstrate differences between CUS and CAS cultured fibroblasts, and may suggest that, also in vivo, CUS and CAS resident fibroblasts may degrade elastic fibers at a different extent, i.e., lower in CUS than in CAS. These results are of particular interest as there is a strong correlation between elastic fiber degradation and mineralization^58,65,66^. Therefore, CUS and CAS fibroblasts, having a different capacity to secrete proteases and their inhibitors, can explain, at least partially, why in PXE not all elastic fibers are calcified even in the same tissue of the same subject.

### Differentially expressed proteoglycans in CAS contribute to ectopic calcification

In the last years it has been demonstrated that proteoglycans, complex macromolecules composed of a core protein to which one or more glycosaminoglycans are covalently bound, play a key role in elastic fiber assembly (e.g., decorin, versican)^67–69^ and also in ectopic calcification (e.g., decorin, biglycan, versican)^70–73^.

We quantified 9 proteoglycans by LC-MS/MS (Supplementary Data 1) of which a basement membrane-specific heparan sulfate proteoglycan core protein (also known as perlecan), decorin and fibromodulin were differentially expressed between CUS and CAS (Supplementary Data 2).

**Fig. 5:**
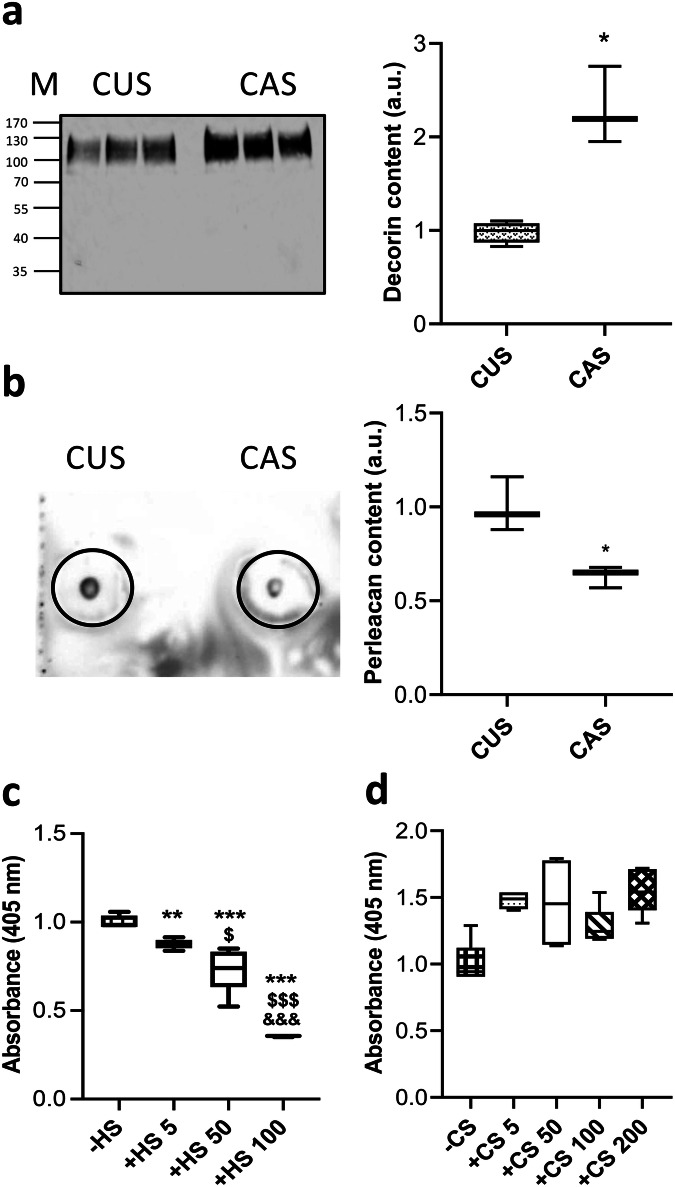
Detection of differentially expressed proteoglycans and mineral deposition. **a** Representative WB of decorin in media conditioned by CUS or CAS fibroblasts (M molecular weight; kDa) and quantification of decorin expression based on WB. Total protein expression served as loading control (Supplementary Fig. 1a, b). Data represent means ± SD of measures from two independent experiments performed in triplicate on cell lines kept separate (*n* = 3 biological replicates for CUS and *n* = 3 biological replicates for CAS). The level of decorin in medium conditioned by CUS fibroblasts was set to 1. *p = 0.0137. **b** Representative dot-blot of perlecan in media conditioned by CUS or CAS fibroblasts (Supplementary Fig. 1c). Data represent means ± SD of measures from two independent experiments performed in triplicate on cell lines kept separate (*n* = 3 biological replicates for CUS and *n* = 3 biological replicates for CAS). The level of perlecan in medium conditioned by CUS fibroblasts was set to 1. *p = 0.015. **c**, **d** Alizarin red assay on insoluble elastin fibrils coacervated in the absence (−) or in the presence (+) of HS (from 5 to 100 μg) or of CS (from 5 to 200 μg), digested with medium conditioned by CAS fibroblasts and incubated with calcifying medium for 72 hours. Data are from three independent experiments performed in technical triplicates (*n* = 3 for each experimental condition). Calcium level of ELN fibrils coacervated without HS or without CS was set to 1. The boxplots show the median (center line), interquartile range (bounds of box), the range of typical data values (whiskers). **p < 0.01; ***p < 0.001 ELN + HS vs. ELN; ^$^*p* < 0.05; ^$$$^*p* < 0.001 50 or 100  μg HS vs 5 μg HS; ^&&&^*p* < 0.001 100 μg HS vs 50 μg HS.

Decorin is a chondroitin/dermatan sulfate-rich proteoglycan involved in elastic fiber assembly^67^ and in collagen fibrillogenesis with different effects depending on the contribution of other molecules and on the physicochemical conditions such as temperature, pH, and ionic strength^74^. Interestingly, decorin has been shown to be upregulated in fibroblasts from PXE patients compared to cells from healthy subjects^75,76^ and in calcifying cultures of aortic SMC, its overexpression/supplementation strongly increasing the calcification process^77^ possibly due to a direct binding of the proteoglycan to hydroxyapatite^78^. Consistently, present data revealed that decorin was upregulated in CAS compared to CUS secretome (Fig. 5a, Supplementary Data 2, and Supplementary Fig. 1a, b).

In contrast, fibromodulin, a keratan sulfate proteoglycan, was significantly downregulated in CAS *vs* CUS secretome. Loss of this molecule has been shown to increase the diameter of collagen fibrils and to impair dentin mineralization depending on the developmental stage in the mouse model^79^ and with opposing effects in the alveolar bone^79^ or in tendons^80^. To our knowledge there are no data concerning the effect of fibromodulin on dermal or on vascular calcification, therefore present data could open the way to additional studies to better understand the possible role of fibromodulin in the context of PXE.

Similarly, perlecan, a secreted heparan sulfate (HS) proteoglycan, was significantly reduced in CAS- compared to CUS secretome (Fig. 5b, Supplementary Fig. 1c and Supplementary Data 2). This proteoglycan was shown to be reduced in calcified arteries^81^ and to be involved in the formation/assembly of elastic fibers^67,82–84^ through the interaction between perlecan-HS chains and tropoelastin^85^ and/or fibrillin 1^83,86–88^. Since perlecan plays a key role in elastic fiber stability, reduced perlecan availability in CAS may lead to the increased susceptibility of elastic fibers to proteolysis that is significantly higher in CAS- compared to CUS secretome.

Since the physical proprieties of proteoglycans are due to glycosaminoglycan chains, we investigated whether addition of different concentrations of glycosaminoglycans had effects on ELN calcification. For this purpose, we used an in vitro model allowing to evaluate the propensity of elastin per se to calcify, independently from the presence of cells. Indeed, ELN fibrils were coacervated in the absence or in the presence of different concentrations (from 5 to 200 μg) of HS or of chondroitin sulfate (CS), two glycosaminoglycans known to interact with elastic fiber^83^ and/or to affect proteinase activity^89,90^.

CS and HS did not affect the size of coacervated ELN fibrils, as observed by light microscopy, except for HS at higher concentration, that determined shorter ELN fibrils (Supplementary Fig. 2) and therefore this concentration was not tested in further experiments. The fact that, at the highest concentration, CS does not have the same effect of HS may be due not only to charge interactions but also to the sugar moieties present in HS that influences ELN coacervation^91^.

ELN fibrils, with or without HS, were digested with the CAS secretome and then incubated with a calcifying medium. Alizarin red assay showed that calcium deposition significantly increased with the decrease of HS concentration in ELN fibrils (Fig. 5c), thus demonstrating that HS protects elastin from calcification.

These data support the hypothesis that also in vivo changes in HS proteoglycans, such as perlecan, may contribute to modulate the mineralization of elastic fibers.

The same type of experiments was performed with CS, but Alizarin red staining did not reveal any significant difference in mineral deposition on ELN fibrils with or without CS (Fig. 5d). Although CS was shown to participate in biomineralization and to promote osteoblasts’ differentiation, the involvement of CS in the calcification process is still controversial^92^. Indeed, some studies indicate that CS promotes collagen calcification and apatite nucleation^92^, whereas, according to other Authors, this glycosaminoglycan inhibits the mineralization process^93,94^.

It is well known that glycosaminoglycans can regulate the self-assembly of tropoelastin, the precursor of elastin^83^ and that both HS and CS can modulate tropoelastin coacervation, however the effects of HS are more significant compared to those of CS^86^. Results from the present study underline the role of HS in the assembly of elastin fibrils and indicate that the presence of HS in the fibrils protects elastin from proteolysis and, therefore, from increased calcification.

It is well known that the composition of the ECM is highly variable depending on the functional requirement of the tissue or of specific areas within the same tissue^95,96^. Indeed, among matrix components, proteoglycans exhibit the highest variability depending on the developmental stage, on the organ/tissue considered, on the cell type involved in their synthesis, and on post-translational modifications^97,98^. Therefore, in the light of present data, it could be suggested that local changes in the expression of glycosaminoglycans/proteoglycans create either a “protective environment” or a “pro-osteogenic milieu”, thus explaining the presence of calcified elastic fibers in specific areas.

### Differentially expressed proteins related to nuclear factor kappa B (NF-kB) pathway

Among DEPs we have also demonstrated the up-regulation of galectin-3, and of high-mobility group box1 (HMGB1) in CAS compared to CUS (Supplementary Data 2).

Galectin-3, a member of the β-galactoside-binding lectin family, is expressed in various tissues exerting multiple functions^99^, including the control of cell-matrix interactions^100^ and the osteogenic differentiation of vascular SMC and of valve interstitial cells^101,102^. Indeed, galectin-3 pharmacological inhibition blocks aortic valve calcification^103^. Some studies demonstrated that galectin-3 regulated calcification by modulating NF-kB, ERK1/2, or Wnt/β-catenin signaling pathways depending on the cell type or tissue^101,102,104,105^.

**Fig. 6:**
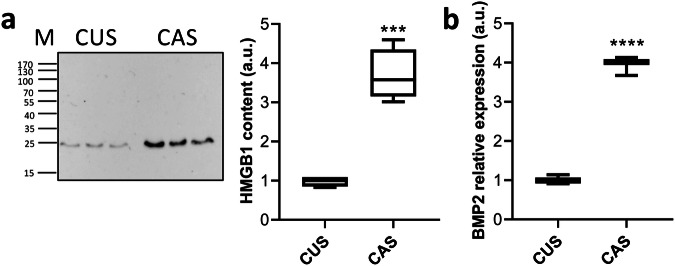
Evaluation of HMGB1 and BMP-2 expression. **a** Representative WB of HMGB1 in media conditioned by CUS or CAS fibroblasts (M molecular weight; kDa) and quantification of HMGB1 expression based on WB. Total protein expression served as loading control (Supplementary Fig. 1d, e). Data represent means ± SD of measures from two independent experiments performed in triplicate on cell lines kept separate (*n* = 3 biological replicates for CUS and *n* = 3 biological replicates for CAS). The level of HMGB1 in medium conditioned by CUS was set to 1. ***p = 0.0002. **b** The mRNA level of *BMP-2* in CUS and CAS fibroblasts was assessed by qRT-PCR. Data represent means ± SD of measures from three independent experiments performed in triplicate on cell lines kept separate (*n* = 3 biological replicates for CUS and *n* = 3 biological replicates for CAS). Expression of *BMP-2* mRNA in CUS fibroblasts was set to 1. ****p < 0.0001. The boxplots show the median (center line), interquartile range (bounds of box), and the range of typical data values (whiskers).

By LC-MS/MS and WB, HMGB1 was significantly increased in CAS compared to CUS (Fig. 6a, Supplementary Data 2 and Supplementary Fig. 1d, e). HMGB1 is a nuclear DNA-binding protein, but it can be secreted in the extracellular space, where it is able to interact with different receptors and interactors to mediate cell proliferation, differentiation, and migration. Moreover, HMGB1 plays an active role in inflammation, bone remodeling, and EC^106^. Wang and collaborators^107^ demonstrated that HMGB1 activated NF-kB pathway inducing the up-regulation of pro-osteogenic molecules and calcium deposition.

In light of these data, it can be suggested that galectin-3 and HMGB1 activate the transcription factor NF-kB, which, in turn, regulates BMP-2, a member of the TGF-β superfamily essential for osteogenesis and for ectopic calcification^108,109^. We have therefore assessed mRNA levels of *BMP-2* in CUS and CAS fibroblasts by qRT-PCR. It has been previously demonstrated that BMP-2 was significantly upregulated in PXE fibroblasts compared to healthy cells^19^. Present data show that CAS fibroblasts have a significantly increased *BMP-2* mRNA expression when compared to CUS cells (Fig. 6b). Since calcification is known to be associated with BMP-2 pathway activation^109,110^, it can be hypothesized that in vivo mineral deposition could be dependent on the level of BMP-2, as observed in cultured human SMC^111^.

Interestingly, both galectin-3 and HMGB1 are known to be related to clinical complications in aging, in diabetes and in chronic kidney disease, which are characterized by high propensity to EC^112–114^. Therefore, galectin-3 and HMGB1 could be new targets for therapeutic intervention against ectopic calcification, as already suggested for age-related diseases^114^.

## Conclusions

Strengths of the study include the comparison of the secretome of fibroblasts from clinically affected (i.e., calcified) and non-affected (i.e., non-calcified) skin biopsies from the same PXE patients, thus avoiding inter-individual variability, and allowing to disclose factors capable to modulate local mineral deposition despite the widely spread effect of circulating pro/anti-calcifying factors associated with the genetic condition. Moreover, LC-MS/MS was used to identify and quantify a large number of molecules released by fibroblasts in the culture medium, and proteomic data were integrated with those from morphological and ultrastructural evaluations (i.e., light and confocal microscopy, scanning electron microscopy), zymography, qRT-PCR and in vitro tests.

However, the study has some limitations: (i) the low number of PXE patients, although it is worth remembering that PXE is a rare genetic disease, and it is not always possible to obtain biopsies from affected, and from unaffected skin of the same patient; (ii) in vitro experiments do not recapitulate the complexity of the natural in vivo environment.

**Fig. 7:**
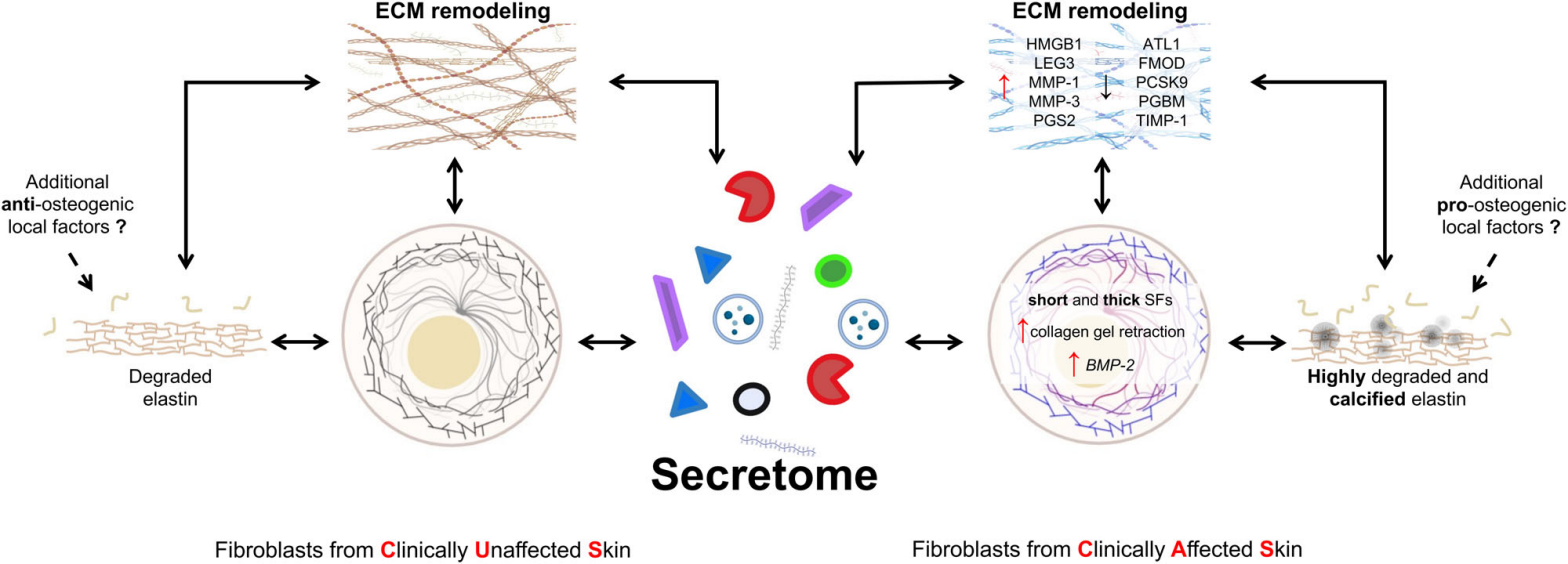
Secretome and ECM remodeling. The drawing highlights the role of fibroblasts’ secretome and of the ECM remodeling in the calcification process. Created with BioRender.com. ATL-1 ADAMTS-like protein 1, *BMP-2* bone morphogenetic protein 2, FMOD fibromodulin, MMP-1 interstitial collagenase, MMP-3 stromelysin-1, HMGB1 high-mobility group protein B1, LEG3 galectin-3, PCSK9 proprotein convertase subtilisin/kexin type 9, PGBM basement membrane-specific heparan sulfate proteoglycan core protein or perlecan, PGS2 decorin, SFs stress fibers, TIMP-1 metalloproteinase inhibitor 1.

Data indicate that cultured CUS and CAS fibroblasts exhibit different cell-matrix interactions and a modified organization of focal adhesions and of stress fibers. These changes are known to directly influence ECM remodeling and/or to affect transcriptional and translational regulation^115,116^. Consistently, cells synthesize and secrete in the extracellular space differentially expressed molecules (e.g., MMPs, metalloproteinase inhibitors, decorin, perlecan, galectin-3 and HMGB1) that contribute (i) to locally modify the extracellular environment, (ii) to alter the signals provided to cells by the extracellular compartment and consequently (iii) to create a microenvironment capable to favor or to inhibit the calcification process (Fig. 7).

In particular, altered stability of elastic fibers leads to fibers more susceptible to proteolysis. Degradation on one side exposes an increased number of calcium binding sites favoring mineral deposition, on the other side releases peptides/elastokines capable to stimulate a pro-osteogenic phenotype^72,117,118^. Mineral deposition, in turn, influences tissue stiffness and the different response of cells to local mechanical stimuli^24^.

Although cells and ECM remodeling are further affected by aging, oxygen availability, hormonal status, mechanical stress^27,119^, nevertheless, this study provides evidence that differentially expressed matrix and matrix-associated molecules are important factors in the development of ectopic calcification. Indeed, differentially expressed secreted proteins guide the level of ECM remodeling to an extent that may lead to degradation (in CUS) or to degradation and calcification (in CAS).

## Methods

### Cell culture

Human dermal fibroblasts were selected within the cryo-stored cell collection of the laboratory. Since females are more frequently affected by PXE compared to males (ratio 2:1)^120^, fibroblasts were isolated from clinically unaffected (arm = absence of papules and/or laxity) and affected skin (axilla = presence of papules and/or laxity) (Table 2) of three Caucasian Italian female patients affected by PXE (44 ± 10 yrs) after informed consent in accordance with the World Medical Association’s Declaration of Helsinki. From each bioptic sample, fibroblast cultures were established^121^, and were grown in 75 cm2 flasks (Nunc, Roskilde, Denmark) in Dulbecco modified eagle medium (DMEM) containing 10% fetal bovine serum^122^. All cell lines were used between the third and the seven passage.

**Table 2:** Clinical characteristics of PXE patients

Phenodex-FlorMore							
Patient	Skin	Eye	Gastro intestinal	Vascular	Cardiac	Renal	
#1	3	4	0	0	0	0	
#2	2	4	0	2	1	0	
#3	3	6	0	0	0	0	

Axillary and arm areas show typical skin papules and laxity (CAS=clinically affected skin) and absence of skin alterations (*CUS* clinically unaffected skin), respectively. The Phenodex-FlorMore scoring system for the evaluation of PXE clinical manifestations. Image is published with the informed consent of the patient.

PXE diagnosis was made based on the phenotype (e.g., skin, ocular, and cardiovascular involvement) (Table 2) and molecular analysis of the *ABCC6* gene (Table 3). All patients were compound heterozygous for rare pathogenic sequence variants (Table 3) already reported^120^.

**Table 3:** Genetic characteristics of PXE patients

*ABCC6* gene mutation				
Patient	Intron/Exon	Nucleotide/Amino Acid variation	Intron/Exon	Nucleotide/Amino Acid variation
#1	19	c.2458 G > C; p.Ala820Pro	IVS26	c.3736-1 G > A
#2	23	c.3088 C > T; p.Arg1030*	IVS17	c.2248-2_2248-1delAG
#3	12	c.1553 G > A; p.Arg518Gln	24	c.3421 C > T; p.Arg1141*

This study was approved by the Ethical Committee of the Faculty of Medicine of the University of Modena (protocol code #47/98 and #136/05) and all ethical regulations relevant to human research participants were followed.

Informed consent was obtained from the patient depicted in the image in Table 2 to publish the picture in this publication.

### Morphological analysis by light microscopy

CUS and CAS fibroblasts were seeded on plates and cultured for 24 hours (at sub-confluence). Briefly, cells were fixed in 4% v/v paraformaldehyde in Dulbecco’s phosphate-buffered saline (DPBS) for 10 min, washed twice with DPBS, and stained with 1% toluidine blue^123^. Images were acquired using a Nikon DS-Fi1 (Nikon Corporation, Tokyo, Japan) camera coupled with a Zeiss Axiophot light microscope. Cell shape descriptors were obtained using Image software (v.1.53t) on 90 cells/sample. Area (total area of each cell), perimeter, aspect ratio (the ratio between the major and minor axis of a cell), and circularity [4π (area)/(perimeter^2^)] were calculated.

### Immunofluorescence and morphometric analyses

CUS and CAS fibroblasts (1.5 × 10^3^ cells/well) were seeded on 20 µg/ml collagen-I (A10483, Gibco) coated glass chamber slides. After 24 hours cells were fixed in 4% (v/v) paraformaldehyde in DPBS, permeabilized with 0.5% Tween-20 in DPBS for 20 min at room temperature (RT). Non-specific binding sites were blocked using 5% BSA in DPBS. Then, the cells were incubated overnight with rabbit anti-human paxillin (dilution 1:400, ab32115, Abcam) at 4  °C in a wet chamber. After washing, samples were incubated with the secondary antibody goat anti-rabbit AlexaFluor-594 (dilution 1:1000, ab150080, Abcam) for 45 min in the dark at RT. Nucleus and actin cytoskeleton staining were performed with 300 nM blue, fluorescent 4’, 6-diamidino-2-phenylindole (D1306, Thermo Fisher Scientific, MA, USA) and iFluor-488 conjugated phalloidin (1:1000, ab176753, Abcam), respectively. After a final washing step, the cells were observed with ×63 Plan-Apo oil immersion objective mounted on a Leica SP8 confocal microscope equipped with a white light laser. Shape descriptors of focal adhesion [i.e., area, perimeter, and shape factor, defined as 4π(area)/(perimeter)^2^] and of stress fibers (i.e., length and width) were calculated by ImageJ and by Focal Adhesion and Filament Cross-Correlation Kit (FAFCK), respectively^124^.

### Total RNA isolation and cDNA synthesis

CUS and CAS fibroblasts were homogenized, and RNA was isolated using the Qiagen RNeasy mini-Kit (Qiagen, Valencia, CA, USA) following manufacturer’s instructions. The purity and concentration of RNA were evaluated using a NanoDrop 2000 spectrophotometer (Thermo Fisher Scientific). The quality of RNA extraction was performed by measuring the optical density (260/280) absorption ratio of 1.8-2.2. 100 ng of each sample was used to synthesize cDNA using the High-Capacity cDNA Reverse Transcription Kit (Applied Biosystems, Waltham, MA, USA).

### Quantitative real‐time‐polymerase chain reaction (qRT-PCR)

Quantitative real-time PCR (qRT-PCR) was performed using CFX Connect Real-Time PCR detection system (Bio-Rad, Hercules, CA, USA). Each qRT-PCR sample was run in triplicate using TaqMan Universal Master MIX II (UNG) supplied by Applied Biosystems with specific TaqMan probe. Each reaction required 2.0 μL cDNA, 10 μl TaqMan Universal Master Mix II, 1 μl TaqMan probe, and 7 μl DNAse-RNase free water. After UNG incubation for 2 min at 50 °C and 10 min at 95 °C for polymerase activation, the qPCR protocol involves 40 cycles of denaturation (95 °C, 15 s), annealing, and elongation (60 °C 1 min). qRT-PCR was used to assess the expression of paxillin and *BMP-2*.

Before performing the paxillin and *BMP-2* gene expression analysis, we identified the reference genes suitable for our experimental conditions, since it is kwon that most of common reference genes are not expressed consistently in all cells under some circumstances^125^. We evaluated the stability of eleven candidate reference genes (i.e*., 18* *S*; *ACTB*; *B2M*; *GAPDH; HMBS; HPRT1; RPL13A; SDHA; TBS; UBC* and *YWHAZ*) in CUS and CAS conditions (Supplementary Fig. 3 and Supplementary Table 1). The stability of this gene panel was investigated using web-based tool RefFinder^126^ integrates four currently available computational programs: NormFinder^127^; geNorm^125^; Dela Cq^128^; BestKeeper^129^ (Supplementary Fig. 4). Under our experimental conditions *RPL13A* and *SDHA* gene expressions were the most stable and therefore used as internal controls for normalization^125^. The relative expression of paxillin and of *BMP-2* was calculated applying the (2^−∆∆Ct^) method.

### Collagen gel retraction

Collagen gels (2 mg protein/mL) were prepared using collagen solution (A10483, Thermo Fisher Scientific) neutralized by adding 0.1 M NaOH. Cells were added at a concentration of 10^5^ cells/mL. The cell/collagen mixture was placed in 12 well plate and left to polymerize for 15 min at 37 °C. To create floating collagen gels, the edge of lattices was rimmed with a sterile pipette. Floating gels were covered with standard culture medium. Gel retraction was evaluated measuring the gel area within 48 hours using ImageJ.

### Preparation of secretome

Fibroblasts were cultured until 80% confluence, then cellular monolayer was washed three times with DPBS to remove FBS and incubated with the same culture medium without FBS for additional 24 hours. The culture medium was collected, centrifuged at 1200 × *g for* 10 min at RT to remove all cellular debris and apoptotic bodies, then concentrated by ultrafiltration using Vivaspin-20 centrifugal filter device in polyethersulfone (nominal molecular weight limit, 10 kDa, Sartorius). The protein concentration was determined by the Bradford method^130^.

### Protein digestion using filter-aided sample preparation (FASP) protocol

50 μg of proteins were reduced in 5 mM 1,4-dithioerythritol (DTE, Merck-Life Science, Darmstadt, Germany) at 60 °C for 30 min. Protein samples were combined with 200 μL of 8 M urea in 100 mM ammonium bicarbonate (AMBIC, pH 8.0), transferred to FASP filters and centrifuged for 20 min at 14,000 × *g*. Proteins were alkylated adding 100 μL of 15 mM iodoacetamide (IAA, Merck-Life Science) in 8 M urea. The membrane was washed 3× with 100 μL of 8 M urea for 20 min at 14,000 × *g*, then twice with 100 μL of 50 mM AMBIC centrifuging at 14,000 × *g* to remove the urea. Digestion was performed in 50 mM AMBIC containing 1 μg of trypsin overnight at 37  °C. Peptides were collected from the membrane by centrifuging for 20 min at 14,000 × *g* and samples were dried using a vacuum centrifuge.

### Liquid chromatography with tandem mass spectrometry (LC-MS/MS) and data analysis

Peptides were resuspended in water/formic acid solution (95:3:2). A UHPLC ultimate 3000 system coupled online to a Q Exactive Hybrid Quadrupole-Orbitrap Mass Spectrometer (Thermo Fisher Scientific) was used^43^. All samples were run in technical quadruplicate in two independent experiments.

MS/MS ions search was performed using Comet search engine (v. 2021.01 rev. 0) integrated in Tran-Proteomic Pipeline (v. 6.0.0) converting raw MS/MS using default settings of msConvert ProteoWizard (v.3.0.1908) to MZML file^123^. Human reference protein datasets were downloaded from Uniprot (UP000005640), integrated with common serum contaminants (cRFP)^131^.

Sequences were reversed to generate decoy database. The selected parameters for protein identification were: (i) at least 1 unique peptide; (ii) static modification carbamidomethyl on cysteines (+57.021 Da), dynamic modifications oxidation on methionine and on proline (+15.995 Da), lysine oxidation to allysine (−1.0316 Da)^58,132^ and deamidation on asparagine and glutamine (+0.984 Da); (iii) precursor mass tolerance of 10 ppm, fragment mass tolerance of 0.02 Da; (iv) the maximum of missed trypsin cleavage sites of 1; (v) the minimum peptide length of 7; (vi) peptides were analyzed by PeptideProphet and the output refined using iProphet excluding number of sibling peptides (NSP) model; (vii) protein validation was performed using ProteinProphet on iProphet output^123^. Briefly, iProphet peptide results (.pepxml) were imported in Skyline-daily (v.21.0.9.139)^43^, to generate spectral libraries. Parameters settled were: (i) 0.99 as spectra cut-off score; (ii) precursor ion charge 2+, 3+, 4+; (iii) MS1 filters were set to “use high selectivity extraction” with a resolving power of 60,000 at 300 m/z; (iv) repeated and duplicate peptides were removed; (v) Fasta files containing proteins with 1% FDR were imported to Skyline to maintain and fix FDR; (vi) only proteins with at least two peptides were considered for quantitative analysis to avoid incorrect quantification across LC-MS runs^123,133^. Label-free quantification was performed using the Equalize medians normalization method and selecting high quality features. Proteins with log_2_ fold change ± 0.58 and an adjusted *p* values < 0.05 were considered as differentially expressed proteins (DEPs)^123^.

To verify the enrichment of the sample with secreted proteins, five databases were utilized: SignalP 6.0 (http://www.cbs.dtu.dk/services/SignalP/), which predicts the presence of signal peptides^134^, UniProtkB (secreted) (https://www.uniprot.org/), protein list located outside the cell membrane(s), Vesiclepedia (http://www.microvesicles.org) contains a list of proteins, lipids, RNA and metabolites identified in extracellular vesicles^135,136^, ExoCarta (http://www.exocarta.org/), an exosome database that provides with the contents identified in exosomes, and MatrisomeDB^2.0^ (https://matrisomedb.org/), a database that includes all structural ECM components and proteins that may directly or indirectly interact with the ECM^43,95^. Moreover, PantherDB (v. 17.0)^137^ was used to annotate protease or protease inhibitors identified in CUS and CAS secretomes.

### Substrate zymography

For each sample, 15 μg of proteins/lane underwent electrophoresis at 4 °C under non-reducing conditions using a 7% SDS-PAGE containing 1% (w/v) collagen. After electrophoresis, gels were washed twice with renaturing solution [2.5% (v/v) Triton-X100 in 50 mM Tris-HCl (pH 8)] for 30 min at room temperature (RT). Gels were incubated in developing buffer (50 mM Tris-HCl, 100 mM NaCl, 10 mM CaCl2, pH 8.0) at 37 °C overnight and then immersed into 0.1% Coomassie Brilliant Blue in 45% (v/v) methanol and 10% (v/v) acetic acid and then incubated with a solution acetic acid, ethanol, and water (10:45:45). Collagenolytic activities were visualized as clear bands over blue background. Quantification of proteolytic activities on zymograms was performed by densitometry scanning and computer-assisted image analysis using the ImageJ software (v.1.53t).

### Elastin fibrillar structure

Insoluble elastin (ELN) (1 mg/mL in ddH_2_O_2_) (Merck-Life Science; E-1625) was placed into sterile tubes at 37 °C for 72 hours to induce the hydration of ELN fibrillar structures. Fibrils were then incubated with standard culture medium (DMEM) or with CUS or CAS conditioned media (i.e., media containing fibroblasts’ released proteases) for 72 hours at 37 °C with 5% CO_2_, then these suspensions were used for coating, with 1 mL, 60 mm diameter plates. Plates were left to dry at RT, and the morphology of elastin fibrils observed with a Zeiss Axiophot light microscope and with a scanning electron microscope (FEI NOVA NanoSEM 450).

For the characterization of the surface of undigested and digested ELN fibrils, root mean square (RMS) roughness of the peaks and valleys were assessed using the SurfCharJ 1q plugin^138^ integrated in ImageJ.

### Alizarin red staining (ARS)

Alizarin red staining was performed on ELN fibrils treated with DMEM or with conditioned media (see previous paragraph for details) and on ELN fibrils coacervated in the absence or in presence of different concentration of heparan sulfate (HS, Opocrin, Corlo-Italy) or of chondroitin sulfate A (Merck-Life Science; C9819) (i.e., 5, 50, 100, and 200 μg). Afterwards, 2 mL of DMEM without phenol red (Thermo Fisher Scientific) and fortified with Pi were added to each plate^58^. Incubation time was set at 72 hours, then plates were washed twice with PBS, fixed with 4% formaldehyde for 15 minutes at RT, washed again with ddH_2_O_2_ and stained with 40 mM ARS (pH 4.2) for 20 min at RT. After washing with ddH_2_O_2_, the dye was extracted, and solution read at 405 nm with a Multiskan FC Microplate Reader (Thermo Fisher Scientific) in 96 well plates. ARS concentration was determined by using a standard curve^139^.

### Dot-blot and western blot (WB)

For perlecan quantitation, dot-blot was performed using 5μg of secretoma proteins in 2 μL of TRIS applied directly onto the nitrocellulose membrane (NcM) that was left to dry at RT for 30 minutes. The NcM was then rinsed with TBST (150 mM NaCl, 50 mM Tris, pH 7.5 plus 0.1% Tween-20) three times and blocked with 5% nonfat milk in TBST for 2 hours. The NcM was incubated with primary antibody (mouse anti-perlecan diluted 1:500; Thermo Fisher Scientific, cat n. 13-4400) for two hours at RT, then washed three times with TBST and incubated with the horseradish peroxidase-conjugated secondary antibody (sheep anti-mouse IgG diluted 1:5000; Abcam ab6808) for 30 minutes at RT. After washing with TBST, the NcM was treated with the ECL substrate solution for 5 minutes and inserted into the Amersham Imager 680 RGB (GE Healthcare) for chemiluminescence analysis. Control of the reaction comprised blots without the step of the incubation with primary antibody to check for non-specific secondary antibody binding to the sample.

For decorin and for HMGB1 quantitation, WB was performed using 20 μg of secretome proteins subjected to a denaturation step with Laemmli buffer, loaded on an 8% or 10% SDS-gel, respectively, and then transferred on NcM. Membranes were blocked in TBST + 5% nonfat dry milk for 1 hour at RT. Primary antibodies were diluted in TBST as follows: rabbit anti-decorin (dilution 1:1000; Abcam ab277643) and rabbit anti-HMGB1 (dilution 1:10000; Abcam ab79823). After washing in TBST, NcMs were incubated with a secondary antibody (donkey anti-rabbit IgG diluted 1:5000; Abcam ab6802). Western blots were visualized using the Amersham Imager 680 RGB (GE Healthcare) according to the manufacturer’s protocols. All WBs were stained with Ponceau S. Protein bands detected on WB by Ponceau S total protein staining or by antibody were quantified using ImageJ and then antibody signals were normalized with total protein stain^140^.

### Statistics and reproducibility

Comparisons of data from the two groups (i.e., CUS and CAS) were done using Mann‒Whitney test. One-way ANOVA followed by Tukey’s posthoc analysis was used for multiple-group comparisons. Statistical analyses were performed using GraphPad Prism 8.0 software (San Diego, CA). Differences were considered significant for *p* < 0.05. Data are reported as boxplots showing the median (center line), interquartile range (bounds of box), the range of typical data values (whiskers). The number of biological replicates and the number of independent experiments are reported in figure legends.

### Reporting summary

Further information on research design is available in the Nature Portfolio Reporting Summary linked to this article.

### Supplementary information

Supplementary Information

Description of Additional Supplementary Files

Supplementary Data 1

Supplementary Data 2

Supplementary Data 3

Supplementary data 4

Reporting summary

## Data Availability

The authors confirm that all the data supporting the findings of this study are available within the article and its Supplementary Data and Supplementary Information. Uncropped blots are shown in Supplementary Fig. 1. The source data behind the graphs in the main and Supplementary Figs. can be found in the Supplementary Data 4. The mass spectrometry proteomics data have been deposited to the ProteomeXchange Consortium via the PRIDE partner repository with the dataset identifier PXD048829.
