# Supplementary material for: Fibroblasts’ secretome from calcified and non-calcified dermis in Pseudoxanthoma elasticum differently contributes to elastin calcification
Source: Commun Biol. 2024 May 16;7:577. doi: 10.1038/s42003-024-06283-6 (PMC11099146; doi:10.1038/s42003-024-06283-6)
Supplement: Supplementary file 1 — Supplementary Information [file 42003_2024_6283_MOESM1_ESM.pdf]

Supplementary Information

Fibroblasts' secretome from calcified and non-calcified dermis in *Pseudoxanthoma elasticum* differently contributes to elastin calcification.

Francesco Demetrio Lofaro, Sonia Costa, Maria Luisa Simone, Daniela Quaglino*, Federica Boraldi*

Department of Life Sciences, University of Modena and Reggio Emilia, Modena, Italy

*Corresponding authors:
daniela.quaglino@unimore.it
federica.boraldi@unimore.it

This file includes:

Supplementary Table 1 List of a) candidates for reference genes and of b) target genes.

Supplementary Figure 1 Uncropped immunoblot images in CUS and CAS secretome for decorin, perlecan and HMGB1.

Supplementary Figure 2 Representative light microscopy image of insoluble elastin coacervated in presence of 200 μg heparan sulphate.

Supplementary Figure 3 Flow chart for the identification of reference genes.

Supplementary Figure 4 Gene stability rankings derived from four computational programs.

Supplementary Table 1. List of a) candidates for reference genes and of b) target genes.

Gene acronymous	Gene name	Gene function	Gene ID	Code HS (ThermoFisher Scientific)
**a) candidates for reference genes**				
**18S**	RNA, 18S ribosomal 1	Essential component of 18S ribosomal subunit.	106632259	Hs99999901_s1
**ACTB**	β-actin	Major constituent of the contractile apparatus.	60	Hs01060665_g1
**B2M**	β-2-microglobulin	Serum protein associated to heavy chain of histocompatibility complex.	567	Hs00187842_m1
**GAPDH**	Glyceraldehyde-phosphate dehydrogenase	Enzyme in glycolysis and gluconeogenesis.	138400	Hs02786624_g1
**HMBS**	Hydroxymethylbilane synthase	Enzyme involved in heme biosynthetic pathway.	3145	Hs00609297_m1
**HPRT1**	Hypoxanthine phosphoribosyl-transferase 1	Enzyme involved in purine synthesis.	3251	Hs02800695_m1
**RPL13A**	Ribosomal protein L13a	Structural component of 60S ribosomal subunit.	40687	Hs 04194366_g1
**SDHA**	Succinate dehydrogenase complex flavoprotein subunit A	Major catalytic subunit of succinate-ubiquinone oxidoreductase, essential complex of the mitochondrial respiratory chain.	6389	Hs07291714_mH
**TBP**	TATA-box binding protein	Component of transcription factor IID (TFIID) is able to bind TATA box.	600075	Hs00427620_m1
**UBC**	Ubiquitin C	Polyubiquitin precursor.	7316	Hs00824723_m1
**YWHAZ**	Tyrosine3-monooxygenase/tryptophan 5-monooxygenase activation protein zeta	Signal transduction	7534	Hs03044281_g1
**b) target genes**				
**BMP2**	Bone morphogenetic protein 2	Growth factor of the TGF-beta superfamily involved in essential processes as cartilage and bone formation.	650	Hs00154192_m1
**PXN**	Paxillin	Cytoskeletal protein that is part of the focal adhesion components.	5829	Hs01104424_m1

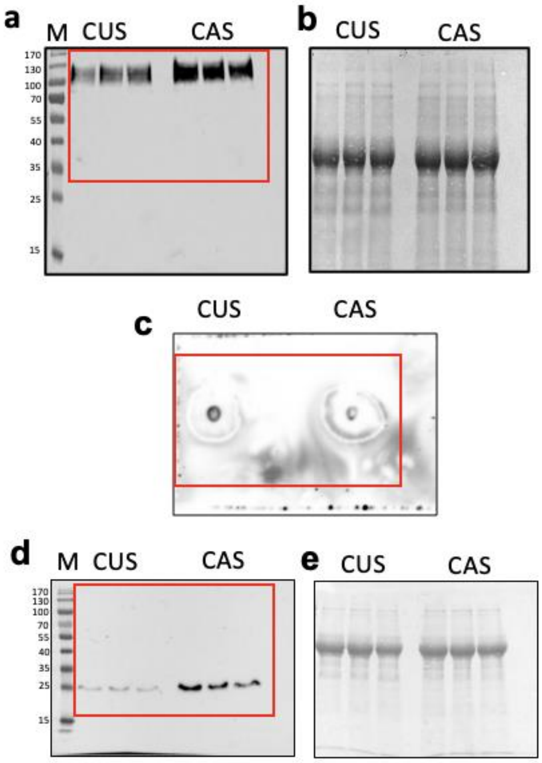

Supplementary Figure 1. Uncropped immunoblot images for decorin, perlecan and HMGB1 in CUS and CAS secretome. Uncropped immunoblot images for decorin protein content **(a)** normalized against the amount the total protein content revealed by Ponceau S **(b)**; for perlecan quantification **(c)**; for HMGB1 protein content **(d)** normalized against the amount the total protein content revealed by Ponceau S **(e)**. The area blot chosen for publication (Figure 5 a, 5b and 6a) is delimited by a red rectangle and the size ladder is aligned with them.

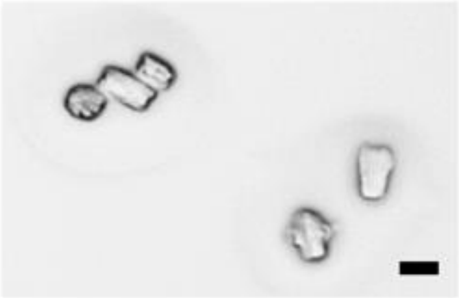

Supplementary Figure 2. Representative light microscopy image of insoluble elastin coacervated in the presence of 200 μg heparan sulphate. Scale bar: 10 μm.

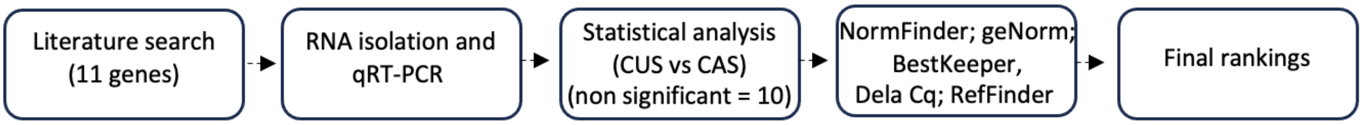

Supplementary Figure 3. Flow chart for the identification of reference genes.

a

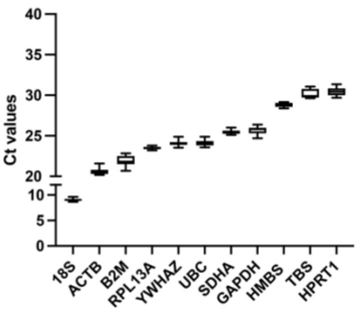

b

BestKeeper	RPL13A	SDHA	GAPDH	HPRT1	HMBS	18S	YWHAZ	UBC	TBS	B2M
n	18	18	18	18	18	18	18	18	18	18
geo Mean [CP]	23.00	25.05	24.99	29.88	28.33	8.60	23.61	23.55	29.49	21.27
AR Mean [CP]	23.00	25.06	25.00	29.89	28.33	8.61	23.61	23.56	29.50	21.28
min [CP]	23.00	25.00	24.00	29.00	28.00	8.00	23.00	23.00	29.00	20.00
max [CP]	23.00	26.00	26.00	31.00	29.00	9.00	24.00	24.00	31.00	22.00
std dev [± CP]	0.00	0.10	0.33	0.40	0.44	0.48	0.48	0.49	0.56	0.56

c d e

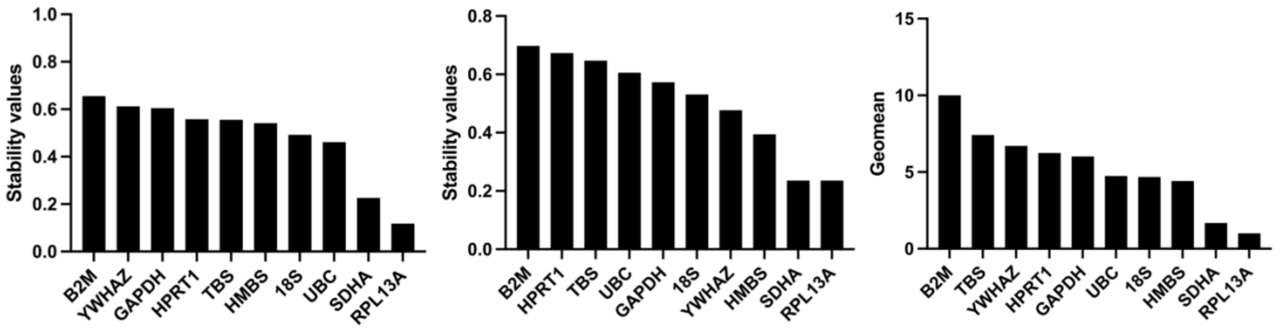

Supplementary Figure 4. a Mean Ct values of each candidate reference gene are shown in a box-and-whisker plot and sorted from the lowest (left) to the highest (right). **b** Bestkeeper candidate reference gene ranking sorted according to their SD and CV% values and their crossing point (CP). **c** NormFinder ranking based on stability value. Candidate reference genes are plotted based on their stability value from the least (left) to the most stable (right). **d** geNorm ranking of stability M value. Candidate reference genes are plotted based on their stability M value from the least (left) to the most stable (right). **e** According to the gene stability rankings derived from four computational programs, RefFinder calculates the geometric mean of weights for the final ranking.
