# Supplementary material for: Fibroblasts’ secretome from calcified and non-calcified dermis in Pseudoxanthoma elasticum differently contributes to elastin calcification
Source: Commun Biol. 2024 May 16;7:577. doi: 10.1038/s42003-024-06283-6 (PMC11099146; doi:10.1038/s42003-024-06283-6)
Supplement: Supplementary file 2 — Description of Additional Supplementary Files [file 42003_2024_6283_MOESM2_ESM.pdf]

Description of Additional Supplementary Files

File name: Supplementary Data 1

Description: List of proteins identified by LCMS/MS in clinically unaffected (CUS) and clinically affected skin (CAS) secretome.

File name: Supplementary Data 2

Description: Label free quantification of proteins identified with at least two peptides in clinically unaffected (CUS) and clinically affected (CAS) skin secretome.

File name: Supplementary Data 3

Description: Protease and protease inhibitors identified in clinically unaffected (CUS) and clinically affected (CAS) skin classified using functional annotation integrated in PantherDB.

File name: Supplementary Data 4

Description: The source data behind the graphs in the main and Supplementary Figures.
