# Supplementary material for: Fibroblasts’ secretome from calcified and non-calcified dermis in Pseudoxanthoma elasticum differently contributes to elastin calcification
Source: Commun Biol. 2024 May 16;7:577. doi: 10.1038/s42003-024-06283-6 (PMC11099146; doi:10.1038/s42003-024-06283-6)
Supplement: Supplementary file 7 — Reporting summary [file 42003_2024_6283_MOESM7_ESM.pdf]

nature portfolio

Corresponding author(s): Federica Boraldi
Daniela Quaglino

Last updated by author(s): Apr 18, 2024

# Reporting Summary

Nature Portfolio wishes to improve the reproducibility of the work that we publish. This form provides structure for consistency and transparency in reporting. For further information on Nature Portfolio policies, see our Editorial Policies and the Editorial Policy Checklist.

## Statistics

For all statistical analyses, confirm that the following items are present in the figure legend, table legend, main text, or Methods section.

| n/a | Confirmed | |
|---|---|---|
| ☐ | ☒ | The exact sample size ($n$) for each experimental group/condition, given as a discrete number and unit of measurement |
| ☐ | ☒ | A statement on whether measurements were taken from distinct samples or whether the same sample was measured repeatedly |
| ☐ | ☒ | The statistical test(s) used AND whether they are one- or two-sided<br>*Only common tests should be described solely by name; describe more complex techniques in the Methods section.* |
| ☒ | ☐ | A description of all covariates tested |
| ☐ | ☒ | A description of any assumptions or corrections, such as tests of normality and adjustment for multiple comparisons |
| ☐ | ☒ | A full description of the statistical parameters including central tendency (e.g. means) or other basic estimates (e.g. regression coefficient) AND variation (e.g. standard deviation) or associated estimates of uncertainty (e.g. confidence intervals) |
| ☒ | ☐ | For null hypothesis testing, the test statistic (e.g. $F$, $t$, $r$) with confidence intervals, effect sizes, degrees of freedom and $P$ value noted<br>*Give P values as exact values whenever suitable.* |
| ☒ | ☐ | For Bayesian analysis, information on the choice of priors and Markov chain Monte Carlo settings |
| ☒ | ☐ | For hierarchical and complex designs, identification of the appropriate level for tests and full reporting of outcomes |
| ☒ | ☐ | Estimates of effect sizes (e.g. Cohen's $d$, Pearson's $r$), indicating how they were calculated |

*Our web collection on statistics for biologists contains articles on many of the points above.*

## Software and code

Policy information about availability of computer code

| | |
|---|---|
| Data collection | MS/MS ions research was performed using Comet search engine (v. 2021.01 rev. 0) integrated in Tran-Proteomic Pipeline (v. 6.0.0) converting raw MS/MS using default settings of msConvert ProteoWizard (v.3.0.1908) to MZML file. Human reference protein datasets were downloaded from Uniprot (UP000005640), integrated with common serum contaminants (cRFP). MS1-level proteome quantification was provided using Skyline-daily (v.21.0.9.139). |
| Data analysis | The annotation of secreted proteins were performed using five databases: SignalP 6.0 (http://www.cbs.dtu.dk/services/SignalP/), Vesiclepedia (http://www.microvesicles), UniProtkB (https://www.uniprot.org/), ExoCarta (http://www.exocarta.org/) and MatrisomeDB (https://matrisomedb.org/) |

For manuscripts utilizing custom algorithms or software that are central to the research but not yet described in published literature, software must be made available to editors and reviewers. We strongly encourage code deposition in a community repository (e.g. GitHub). See the Nature Portfolio guidelines for submitting code & software for further information.

## Data

Policy information about availability of data

All manuscripts must include a data availability statement. This statement should provide the following information, where applicable:

- Accession codes, unique identifiers, or web links for publicly available datasets
- A description of any restrictions on data availability
- For clinical datasets or third party data, please ensure that the statement adheres to our policy

The mass spectrometry proteomics data have been deposited to the ProteomeXchange Consortium via the PRIDE partner repository with the dataset identifier PXD048829

## Research involving human participants, their data, or biological material

Policy information about studies with human participants or human data. See also policy information about sex, gender (identity/presentation), and sexual orientation and race, ethnicity and racism.

| | |
|---|---|
| Reporting on sex and gender | Skin fibroblasts derived from female patients |
| Reporting on race, ethnicity, or other socially relevant groupings | Caucasian Italian patients |
| Population characteristics | Patients were affected by Pseudoxanthoma elasticum (PXE) |
| Recruitment | Fibroblast were obtained from diagnosed PXE patients after informed consent and cryo-stored in the laboratory |
| Ethics oversight | Ethical Committee of Faculty of Medicine (Modena, Italy) (protocol code #47/98 and #136/05) |

Note that full information on the approval of the study protocol must also be provided in the manuscript.

# Field-specific reporting

Please select the one below that is the best fit for your research. If you are not sure, read the appropriate sections before making your selection.

☒ Life sciences ☐ Behavioural & social sciences ☐ Ecological, evolutionary & environmental sciences

For a reference copy of the document with all sections, see nature.com/documents/nr-reporting-summary-flat.pdf

# Life sciences study design

All studies must disclose on these points even when the disclosure is negative.

| | |
|---|---|
| Sample size | No statistical methods were used to determine sample size. PXE is a rare genetic disease, and human skin samples from different area of the same patient are rarely available. |
| Data exclusions | No data were excluded |
| Replication | Data are from triplicate on all cell lines. Experimental procedures are detailed in the manuscript. |
| Randomization | Allocation was not random because the selection was based on the presence/absence of skin alterations. |
| Blinding | Authors were blinded when possible. |

# Reporting for specific materials, systems and methods

We require information from authors about some types of materials, experimental systems and methods used in many studies. Here, indicate whether each material, system or method listed is relevant to your study. If you are not sure if a list item applies to your research, read the appropriate section before selecting a response.

## Materials & experimental systems

| n/a | Involved in the study | |
|---|---|---|
| ☐ | ☒ | Antibodies |
| ☐ | ☒ | Eukaryotic cell lines |
| ☒ | ☐ | Palaeontology and archaeology |
| ☒ | ☐ | Animals and other organisms |
| ☐ | ☒ | Clinical data |
| ☒ | ☐ | Dual use research of concern |
| ☒ | ☐ | Plants |

## Methods

| n/a | Involved in the study | |
|---|---|---|
| ☒ | ☐ | ChIP-seq |
| ☒ | ☐ | Flow cytometry |
| ☒ | ☐ | MRI-based neuroimaging |

# Antibodies

| | |
|---|---|
| Antibodies used | Antibodies used in this study:<br>-anti-human paxillin (dilution 1:400, ab32115,Abcam)<br>- antibody goat anti-rabbit AlexaFluor-594 (dilution 1:1000, ab150080, Abcam)<br>- mouse anti-perlecan (dilution 1:500; ThermoFisher, cat n. 13-4400<br>- rabbit anti-decorin (dilution 1:000; Abcam ab277643)<br>- rabbit anti HMGB1 (dilution 1:10000; Abcam ab79823)<br>- HRP-conjugated sheep anti-mouse Ig antibody (dilution 1:5000 GE Healthcare)<br>- donkey anti-rabbit IgG (dilution 1:5000; Abcam ab6802) |
| Validation | Antibodies were validated by vendors:<br>https://www.abcam.com/en-it/products/primary-antibodies/anti-paxillin-antibody-e228-ab32115<br>https://www.thermofisher.com/antibody/product/Perlecan-Antibody-clone-7B5-Monoclonal/13-4400<br>https://www.abcam.com/en-nl/products/primary-antibodies/anti-decorin-antibody-epr24097-105-bsa-and-azide-free-ab27764<br>https://www.abcam.com/en-kr/products/primary-antibodies/hmgb1-antibody-epr3507-ab79823 |

# Eukaryotic cell lines

Policy information about cell lines and Sex and Gender in Research

| | |
|---|---|
| Cell line source(s) | Fibroblasts were isolated from clinically unaffected and affected skin of three female PXE patients. |
| Authentication | Standard method for human skin fibroblast isolation was used (Methods Mol Med. 2005;117:83-98, doi:10.1385/1-59259-940-0:083) and cells were stored in the local cell culture facility. |
| Mycoplasma contamination | All cell lines were negatively tested for mycoplasma |
| Commonly misidentified lines (See ICLAC register) | N/A |

# Clinical data

Policy information about clinical studies

All manuscripts should comply with the ICMJE guidelines for publication of clinical research and a completed CONSORT checklist must be included with all submissions.

| | |
|---|---|
| Clinical trial registration | This study is not part of a clinical trial |
| Study protocol | PXE patients belong to the Italian PXE patients' Association, on a voluntary basis after informed consent, provided skin biopsies for the establishment of fibroblasts' cell cultures to be used and cryostored for research on their disease. |
| Data collection | Samples were collected from 1998 until 2008 with the approval of Ethical Committee of the Faculty of Medicine of the University of Modena (protocol code #47/98 and #136/05). |
| Outcomes | Aim of the study was use fibroblasts from patients to better understand the pathogenesis of elastic fiber calcification in PXE |

## Plants

Seed stocks

*Report on the source of all seed stocks or other plant material used. If applicable, state the seed stock centre and catalogue number. If plant specimens were collected from the field, describe the collection location, date and sampling procedures.*

Novel plant genotypes

*Describe the methods by which all novel plant genotypes were produced. This includes those generated by transgenic approaches, gene editing, chemical/radiation-based mutagenesis and hybridization. For transgenic lines, describe the transformation method, the number of independent lines analyzed and the generation upon which experiments were performed. For gene-edited lines, describe the editor used, the endogenous sequence targeted for editing, the targeting guide RNA sequence (if applicable) and how the editor was applied.*

Authentication

*Describe any authentication procedures for each seed stock used or novel genotype generated. Describe any experiments used to assess the effect of a mutation and, where applicable, how potential secondary effects (e.g. second site T-DNA insertions, mosiacism, off-target gene editing) were examined.*
